# De novo genome assembly of a high-protein soybean variety HJ117

**DOI:** 10.1186/s12863-024-01213-1

**Published:** 2024-03-04

**Authors:** Zhi Liu, Qing Yang, Bingqiang Liu, Chenhui Li, Xiaolei Shi, Yu Wei, Yuefeng Guan, Chunyan Yang, Mengchen Zhang, Long Yan

**Affiliations:** 1 Hebei Key Laboratory of Crop Genetics and Breeding, Huang-Huai-Hai Key Laboratory of Biology and Genetic Improvement of Soybean, Ministry of Agriculture and Rural Affairs, Institute of Cereal and Oil Crops, National Soybean Improvement Center Shijiazhuang Sub- Center, Hebei Academy of Agricultural and Forestry Sciences, 050035 Shijiazhuang, Hebei China; 2 College of Life Sciences, Hebei Agricultural University, 071001 Baoding, Hebei China; 3 Guangdong Provincial Key Laboratory of Plant Adaptation and Molecular Design, Innovative Center of Molecular Genetics and Evolution, School of Life Sciences, Guangzhou University, 510006 Guangzhou, Guangdong China

**Keywords:** Soybean, De novo assembly, Genome feature, High protein content

## Abstract

**Objectives:**

Soybean is an important feed and oil crop in the world due to its high protein and oil content. China has a collection of more than 43,000 soybean germplasm resources, which provides a rich genetic diversity for soybean breeding. However, the rich genetic diversity poses great challenges to the genetic improvement of soybean. This study reports on the de novo genome assembly of HJ117, a soybean variety with high protein content of 52.99%. These data will prove to be valuable resources for further soybean quality improvement research, and will aid in the elucidation of regulatory mechanisms underlying soybean protein content.

**Data description:**

We generated a contiguous reference genome of 1041.94 Mb for HJ117 using a combination of Illumina short reads (23.38 Gb) and PacBio long reads (25.58 Gb), with high-quality sequence coverage of approximately 22.44× and 24.55×, respectively. HJ117 was developed through backcross breeding, using Jidou 12 as the recurrent parent and Chamoshidou as the donor parent. The assembly was further assisted by 114.5 Gb Hi-C data (109.9×), resulting in a contig N50 of 19.32 Mb and scaffold N50 of 51.43 Mb. Notably, Core Eukaryotic Genes Mapping Approach (CEGMA) assessment and Benchmarking Universal Single-Copy Orthologs (BUSCO) assessment results indicated that most core eukaryotic genes (97.18%) and genes in the BUSCO dataset (99.4%) were identified, and 96.44% of the genomic sequences were anchored onto twenty pseudochromosomes.

## Objective

Soybean [*Glycine max* (L.) Merr.] is an important protein feed and vegetable oil crop worldwide. The cultivation of soy enables the production of various valuable products, including edible oils, biodiesel, and biofertilizers [1]. The main protein source in poultry and livestock feed is meal derived from soybean seeds. Commercial soybean cultivars generally have a seed protein content ranging from approximately 38–42% on a dry weight basis [2]. Only soybean grains with a protein content of 41.5% or higher on a dry weight basis can be used to produce meal with a protein content of 47.5% or higher [2]. Enhancing the amino acid content of soybean seeds would further increase the economic value of soybean. Soy protein content is influenced by complex factors such as genotype, environment, and genotype–environment interactions [3, 4]. Due to the strong negative correlations of soy protein content and oil content [4] with yield [5], it is quite difficult to increase soy protein content.

In the early stages of soybean breeding, farmers primarily relied on repeatedly selecting preferred seeds from cultivated populations [6]. Following that, artificial hybridization technology was introduced, and the initial artificially hybridized cultivated soybean was introduced in North America during the 1940s [7]. With the development and progress of molecular biology technology, marker-assisted selection (MAS) has been employed to expedite the breeding process [8]. The publication of the initial reference genome of soybean (cultivar Williams 82) in 2010 [9] signaled the commencement of the soybean functional genomics research era [10, 11]. The enhancement of sequencing technologies has significantly boosted the capacity to generate high-quality genome assemblies.

## Data description

The *Glycine max* sample was collected from Shijiazhuang (37°6′25″N, 114°42′47″E). Genomic DNA and total RNA were isolated from leaf tissues. High-quality DNA was extracted using QIAGEN® Genomic kits. Three methods were used to quantify and check the extracted DNA, NanoDrop 2000 Spectrophotometer (Thermo Fischer Scientific), agarose gel electrophoresis and Qubit Fluorometer (Invitrogen). After the detection, the DNA was purified using AMPure PB beads (Pacbio 100-265-900), and the subsequent library construction utilized the final high-quality genomic DNA (gDNA). The size and concentration of the library fragments were assessed using an Agilent 2100 Bioanalyzer (Agilent Technologies, USA). Qualified libraries were evenly loaded on SMRT Cell and sequenced for 30 h using Sequel II/IIe system (Pacific Biosciences, CA, USA).

Briefly, the DNA sample was initially fixed with formaldehyde and subsequently digested using *Hind*III restriction enzyme. Next, the DNA ends underwent repair and were labeled with biotin. Subsequently, T4 DNA ligase was used to ligate the interacting fragments to form a loop. After ligation, protease K was added for cross-linking, and then protein of ligated DNA fragments was digested to obtain purified DNA. Finally, the purified DNA was fragmented into sizes ranging from 300 to 500 base pairs. The biotin-labeled DNA fragments were then isolated using Dynabeads® M-280 Streptavidin (Life Technologies). Subsequently, the Hi-C library was constructed and sequenced on the Illumina NovaSeq6000 sequencing platform using paired-end reads of 150 base pairs.

To ensure the acquisition of high-quality data, the raw polymerase reads were subjected to quality control using the PacBio SMRT-Analysis package (https://www.pacb.com). This involved filtering out the following types of polymerase reads: (1) polymerase reads less than 50 bp in length, (2) Polymerase readings with a mass value below 0.8, (3) a polymerase read comprising an adaptor attached to itself and removing the adaptor sequence in the polymerase read. Then use SMRTLink 9.0 (parameter --min-passes = 3 --min-rq = 0.99) to generate CCS reads for subsequent assembly.

Hifiasm (https://github.com/chhylp123/hifiasm) was employed to assemble the HiFi reads, and the preliminarily assembled genome version (primary contigs) was obtained. To obtain chromosome level genome, we performed Hi-C assisted assembly. For the ~114.5 Gb raw reads (Data file 1 and Data file 2), preliminary quality control was performed using Fastp [14], and the resulting clean reads were subsequently aligned to primary contigs using hicup. Valid pair reads were utilized for further analysis. AllHIC was used for auxiliary assembly, and then Juicebox was used for fine-tune AllHIC clustering results. Finally, A genome was obtained with a contig N50 length of 19.32 Mb and a total contig length of 1041.94 Mb, as well as a scaffold N50 length of 51.43 Mb and a total scaffold length of 1041.95 Mb (Data file 3 and Data file 4).

To assess the quality of the assembly the self-written script was used to perform statistics on the number of single chromosome cluster scaffolds, chromosome sequence length, and genome mounting rate. According to the number of sequences assembled to the chromosome level and the number of sequences that were not assembled to the chromosome level, the Hi-C mounting rate was calculated. The chromosome-level genome was partitioned into 500 Kb bins of equal length. The number of Hi-C read pairs spanning any two bins was used as the intensity signal to represent the interaction between the respective bins. Heatmaps (Data file 5) were generated based on these signals. BUSCO (Benchmarking Universal Single-Copy Orthologs: http://busco.ezlab.org/) [18] was also applied to perform a quality assessment of the genome. The conserved genes (248 genes) existing in six eukaryotes were selected to construct the core gene library for CEGMA [19] evaluation. The evaluation results revealed that the majority of core eukaryotic genes (97.18%) and genes in the BUSCO dataset (99.4%) were successfully identified (Data file 6).

Repeatmasker [21] and repeatproteinmask (http://www.repeatmasker.org/) were employed to identify sequences that exhibit similarity to known repeat sequences. LTR_FINDER [22] was used to perform de novo prediction. Totally, 361,475,923 bp RepBase TEs and 453,714,080 bp de novo repetitive sequences were identified, respectively (Data file 7). Structural prediction of genes was performed by using AUGUSTUS (http://bioinf.uni-greifswald.de/AUGUSTUS/) [24] (Data file 8 and Data file 9). Then, we used the protein databases NR (https://ftp.ncbi.nlm.nih.gov/blast/db/FASTA/), SwissProt (http://www.uniprot.org/), KEGG (http://www.genome.jp/kegg/) and InterPro (https://www.ebi.ac.uk/interpro/) to annotate the gene set obtained from the gene structure annotation. A total of 57,151 genes were predicted, with 54,550 of these genes being functionally annotated in the database (Data file 10). The circular plot illustrates gene density, transposable element (TE) density, and GC density (Data file 11). The tRNAscan-SE [29] (http://lowelab.ucsc.edu/tRNAscan-SE/) was used to identify tRNA sequences within the genome. Blast [30] alignment was used to find the rRNA in the genome. The prediction of miRNA and snRNA sequences within the genome was performed using INFERNAL (http://infernal.janelia.org/). The copy number of miRNA, tRNA, rRNA and snRNA ranged from 68 to 5,116 (Data file 12) (See Table 1).

**Table 1:** Overview of data files/data sets

Label	Name of data file/data set	File type (file extension)	Data repository and identifier (DOI or accession number)
Data file1	Statistics on sequence data	Spreadsheet (.xls)	Figshare,https://doi.org/10.6084/m9.figshare.24865518figshare.com/s/6de11eca18b3ccef8314 [12]
Data file2	Hi-C raw data	Fastq file (.fastq)	NGDC Genome Sequence Archive, https://ngdc.cncb.ac.cn/gsa/browse/CRA014073 [13]
Data file3	Assembly statistics of HJ117	Spreadsheet (.xls)	Figshare,https://doi.org/10.6084/m9.figshare.24865518figshare.com/s/6de11eca18b3ccef8314 [15]
Data file4	genome.fa	Fasta file (.fasta)	NGDC Genome warehouse, https://ngdc.cncb.ac.cn/gwh/Assembly/83716/show [16]
Data file5	Hi-C interaction heatmap	Image file (.tif )	Figshare,https://doi.org/10.6084/m9.figshare.24865518figshare.com/s/6de11eca18b3ccef8314 [17]
Data file6	Assessment results of CEGMA and BUSCO	Spreadsheet (.xls)	Figshare,https://doi.org/10.6084/m9.figshare.24865518figshare.com/s/6de11eca18b3ccef8314 [20]
Data file7	Results of transposable element classification statistics	Spreadsheet (.xls)	Figshare,https://doi.org/10.6084/m9.figshare.24865518figshare.com/s/6de11eca18b3ccef8314 [23]
Data file8	Results of gene structure prediction	Spreadsheet (.xls)	Figshare,https://doi.org/10.6084/m9.figshare.24865518figshare.com/s/6de11eca18b3ccef8314 [25]
Data file9	Glycine.max.gene.gff	Gff file (.gff)	Figshare,https://doi.org/10.6084/m9.figshare.24865518figshare.com/s/6de11eca18b3ccef8314 [26]
Data file10	Genome annotation of HJ117	Spreadsheet (.xls)	Figshare,https://doi.org/10.6084/m9.figshare.24865518figshare.com/s/6de11eca18b3ccef8314 [27]
Data file11	Overview of the HJ117 reference genome	Image file (.tif )	Figshare,https://doi.org/10.6084/m9.figshare.24865518figshare.com/s/6de11eca18b3ccef8314 [28]
Data file12	Statistics on non-coding RNA annotation results	Spreadsheet (.xls)	Figshare,https://doi.org/10.6084/m9.figshare.24865518figshare.com/s/6de11eca18b3ccef8314 [31]
Data file13	Raw RNA reads of leaf tissues	Fastq file (.fastq)	NGDC Genome Sequence Archive, https://ngdc.cncb.ac.cn/gsa/browse/CRA014073 [34]
Data file14	HiFi raw data	Fastq file (.fastq)	NGDC Genome Sequence Archive, https://ngdc.cncb.ac.cn/gsa/browse/CRA014073 [35]

## Limitations

Soybean is considered to have undergone an allotetraploidy event [9] that have resulted in 75% of its genes being present in multiple copies [32]. Repetitive DNA made up ~54.4% of each genome [33]. In this study, 23.38 Gb Illumina short reads (Data file 13) and 25.58 Gb of PacBio long reads (Data file 14) were obtained, providing approximately 22.44× and 24.55× sequence coverage. Although Hi-C sequencing obtained 114.5 Gb of data with a depth of 109.9×, the overall sequencing depth was relatively low, which may result in incomplete genomic information being obtained.

The contig N50 length of the de novo assembled HJ117 genome is 19.32 Mb, and the scaffold N50 reaches 51.43 Mb, indicating that the genome assembly level has achieved the average level of soybean genome assemblies during the same period. However, gaps still exist in the genome. To achieve accurate genome assembly, optical mapping technology could be incorporated, and HiFi sequencing depth could be increased in the later stages. Alternatively, HJ117 genome could be assembled to a telomere-to-telomere level using ONT Ultra-long technology to obtain more comprehensive genomic information for HJ117.

## Data Availability

Data files 2,13,14 described in this Data note can be freely and openly accessed on the Genome Sequence Archive in National Genomics Data Center China National Center for Bioinformation / Beijing Institute of Genomics, Chinese Academy of Sciences under GSA: CRA014073 (https://ngdc.cncb.ac.cn/gsa/browse/CRA014073) [13,34,35]. Data files 4 described in this Data note can be freely and openly accessed on the Genome warehouse in National Genomics Data Center China National Center for Bioinformation / Beijing Institute of Genomics, Chinese Academy of Sciences under GWH: GWHERCR00000000 (https://ngdc.cncb.ac.cn/gwh/Assembly/83716/show) [16]. Data files 1,3,5-12 are available on Figshare (10.6084/m9.figshare.24865518) [12,15,17,20,23,25,26,27,28,31]. Please see Table 1 and references for details and links to the data.

## Abbreviations

CEGMA: Core Eukaryotic Genes Mapping Approach
BUSCO: Benchmarking Universal Single-Copy Orthologs
DNA: Deoxyribonucleic Acid
RNA: Ribonucleic Acid
TE: Transposable Element
Hi-C: High-resolution Chromosome Conformation Capture
HiFi: High-Fidelity Sequencing
HJ117: Ji HuiJiao No.117
